# IVC CLAMP: infrahepatic inferior vena cava clamping during hepatectomy - a randomised controlled trial in an interdisciplinary setting

**DOI:** 10.1186/1745-6215-10-94

**Published:** 2009-10-13

**Authors:** Nuh N Rahbari, Johannes B Zimmermann, Moritz Koch, Thomas Bruckner, Thomas Schmidt, Heike Elbers, Christoph Reissfelder, Markus A Weigand, Markus W Büchler, Jürgen Weitz

**Affiliations:** 1 Department of General, Visceral and Transplant Surgery, University of Heidelberg, Germany; 2 Department of Anaesthesiology, University of Heidelberg, Germany; 3 Institute of Medical Biometry and Informatics, University of Heidelberg, Germany; 4 Department of Anaesthesiology, Justus-Liebig-University Giessen, Germany

## Abstract

**Background:**

Intraoperative haemorrhage is a known predictor for perioperative outcome of patients undergoing hepatic resection. While anaesthesiological lowering of central venous pressure (CVP) by fluid restriction is known to reduce bleeding during transection of the hepatic parenchyma its potential side effects remain poorly investigated. In theory it may have negative effects on kidney function and tissue perfusion and bears the risk to result in severe haemodynamic instability in case of profound intraoperative blood loss. The present randomised controlled trial evaluates efficacy and safety of infrahepatic inferior vena cava (IVC) clamping as an alternative surgical technique to reduce CVP during hepatic resection.

**Methods/Design:**

The proposed IVC CLAMP trial is a single-centre randomised controlled trial with a two-group parallel design. Patients and outcome-assessors are blinded for the treatment intervention. Patients undergoing elective hepatic resection due to any reason are enrolled in IVC CLAMP. All patients admitted to the Department of General-, Visceral-, and Transplant Surgery, University of Heidelberg for elective hepatic resection are consecutively screened for eligibility and written informed consent is obtained on the day before surgery. The primary objective of this trial is to assess and compare the amount of blood loss during hepatic resection in patients receiving surgical CVP reduction by clamping of the IVC as compared to anaesthesiological CVP without infrahepatic IVC clamping reduction. In addition to blood loss a set of general as well as surgical variables are analysed.

**Discussion:**

This is a randomised controlled patient and observer blinded two-group parallel trial designed to assess efficacy and safety of infrahepatic IVC clamping during elective hepatectomy.

**Trial registration:**

ClinicalTrials NCT00732979

## Background

The outcome of patients undergoing hepatic resection has markedly improved since the late 1980's [1]. High-volume centres currently report morbidity and mortality rates of 30 - 45% and 3 - 4% [1-3], respectively and hepatic resections can nowadays be safely carried out without routine use of portal triad clamping with its potential drawback of ischaemia and reperfusion injury [4]. The decrease of intraoperative blood loss and need for blood transfusion, two well-known predictors for the outcome of patients undergoing hepatic resection [3,5], are considered main contributors for the decrease in morbidity and mortality. The observed lower blood loss and transfusion rates most likely result from advances in perioperative care and in particular the reduction of central venous pressure (CVP) during transection of the liver parenchyma. Several non-randomised as well as one randomised trial reported reduction of CVP to result in significantly less intraoperative blood loss [6-8]. For this reason many surgeons currently prefer to perform hepatic resections after previous CVP reduction below 5 mmHg which is usually achieved by restricted intravenous fluid administration, and - if required - the application of diuretics and nitroglycerine [8]. This approach is effective in lowering CVP and by this intraoperative bleeding. However, it bears the risk of various side effects and data on its safety are scarce. Besides impairment of kidney function and tissue oxygenation, hypovolaemic patients are at risk for haemodynamic instability particularly in case of unexpected intraoperative haemorrhage. Moreover, systemic response to restricted volume administration is mostly unknown and requires further research. Based on these potential drawbacks the optimal strategy for the reduction of bleeding during hepatectomy remains a matter of debate.

Selective clamping of the inferior vena cava (IVC) below the liver might serve as an alternative technique to reduce CVP during transection of the liver parenchyma. This approach allows the surgeon to operate under the condition of lowered CVP without the requirement of systemic fluid restriction. The current trial is designed to assess efficacy and safety of selective infrahepatic IVC clamping without previous anaesthesiological CVP reduction as compared to current standard of CVP reduction via fluid restriction and administration of diuretics or nitro-compounds without clamping of the IVC.

### Existing evidence and need for the trial

To assess available evidence, we conducted a systematic literature search of the Medline database according to the criteria of the Cochrane Collaboration (search date: September 4^th ^2008). Furthermore, the reference lists of relevant articles on hepatic vascular control were cross-searched manually for additional studies. The search found several randomised trials evaluating various clamping techniques for reduction of bleeding during hepatectomy [9-13]. Otsubo et al. provided the first report of infrahepatic IVC clamping in a retrospective study of 103 consecutive patients with CVP > 5 mmHg who underwent right or left hemihepatectomy [14]. While this study revealed reduced bleeding for those patients who received IVC clamping below the liver and suggested a positive impact of IVC clamping on bleeding and potentially outcome of patients undergoing hepatectomy, it's retrospective, non-randomised study design does not allow for general recommendation of infrahepatic IVC clamping. Kato et al. published a randomised controlled trial (RCT) evaluating IVC clamping in combination with anaesthesiological CVP reduction in patients undergoing liver resection [15]. The overall design of this trial comparing anaesthesiological CVP reduction to anaesthesiological CVP reduction with additional infrahepatic IVC clamping does not allow for conclusions regarding the treatment effect of infrahepatic IVC clamping in euvolaemic patients. To our knowledge there is currently no RCT available comparing the anaesthesiological CVP reduction (by fluid restriction, use of diuretics, nitro-compounds with no clamping of the IVC) to the surgical CVP reduction (by transient clamping of the IVC below the liver without fluid restriction).

### Aim of this trial

Reduction of blood loss is a major goal in hepatic resections, as major blood loss is a known predictor of patient's outcome [3,5]. Unfortunately, most surgical techniques which aim to decrease bleeding during hepatectomy include portal triad clamping bearing the risk of ischaemic/reperfusion injury to the liver remnant. Reduction of CVP as currently performed may have detrimental effects on tissue perfusion and kidney function and may, moreover, result in severe haemodynamic instability in case of intraoperative haemorrhage. For this reason the present randomised trial evaluates the technique of selective infrahepatic IVC clamping to lower CVP in patients undergoing hepatectomy without routine use of portal triad clamping and without concomitant anaesthesiological CVP reduction. Patients in the control group are treated according to the current local standards consisting of anaesthesiological CVP reduction primarily achieved via fluid restriction and application of diuretics or nitro-compounds without routine use of any kind of vascular clamping.

## Methods/design

### Trial population and patient recruitment

Patients scheduled for elective hepatic resection due to any reason may be enrolled in IVC CLAMP. Table 1 displays the detailed inclusion and exclusion criteria. All patients admitted to the Department of General, Visceral and Transplant Surgery, University of Heidelberg are consecutively screened for eligibility and written informed consent is obtained on the day before surgery.

**Table 1:** Eligibility criteria for IVC CLAMP.

**Inclusion criteria**	**Exclusion criteria**
• Age ≥ 18 years	• Presence of medical conditions putting the individual patient at increased risk for not tolerating liver resection:
• Elective hepatic resection due to any reason	- Liver cirrhosis (Child-Pugh B or C)
• American Society of Anaesthesiologists (ASA) score I to III [26]	- (Hereditary) coagulopathy
• Written informed consent	• Presence of medical conditions putting the individual patient at increased risk of not tolerating at least one of this trial's study interventions:
	- Severe heart disease (e.g. severe coronary artery disease requiring intervention, cardiac insufficiency NYHA stage IV) [16]
	- Pulmonary hypertension
	- Renal insufficiency (serum creatinine > 2 mg/dl or > 177 μmol/l; conversion factor 88.4 or requiring dialysis)
	- Uncorrected electrolyte imbalance
	• Atrial fibrillation
	• For female patients: pregnancy or lactation
	• Technical impossibility of hepatic resection
	• Participation in other clinical trials or observation period of competing trials interfering with the endpoints of this trial
	• Former participation in the clinical trial
	• Suspected lack of compliance
	• Impaired mental state or language problems

### Safety aspects

All hepatectomies within IVC CLAMP are carried out by experienced hepatic surgeons. A recently published article on 300 hepatectomies performed at the Department of General, Visceral and Transplant Surgery, University of Heidelberg using anaesthesiological CVP reduction demonstrated hepatic resections of being safe without routine use of inflow control [2]. Hence, patients randomised to the control group face no additional risk as compared to patients treated at the department outside of the trial.

Infrahepatic IVC clamping lowers the CVP potentially without systemic effects of hypovolemia such as microcirculatory disturbances. It might therefore protect the patient from these unwanted sequelae of hypovolaemia and may improve patient's outcome. Haemodynamic intolerance is, however, a potential side effect of this intervention. For this reason each patient's medical history is scrutinised for the presence of medical conditions putting the individual patient at increased risk of not tolerating the procedure. Patients with severe heart disease, namely severe coronary artery disease (CAD) requiring intervention or cardiac insufficiency (New York Heart Association, NYHA IV [16]) are excluded from the study. To assess patients' hemodynamic tolerance the IVC is clamped for a short test period prior to the actual parenchymal transection. Moreover, patients are monitored very closely by the anaesthetist and the surgeon immediately stops IVC clamping in case it is not tolerated by the individual patient.

For the assessment of safety, adverse events (AE) and serious adverse events (SAE) are documented on separate forms and analysed within the safety analysis. Besides SAEs a set of predefined AEs is documented within IVC CLAMP (Table 2).

**Table 2:** Secondary endpoints of the IVC CLAMP Trial.

**Secondary endpoint**	**Definition and assessment of outcomes**
Blood transfusions:	Administration of PRBC transfusion is documented for the intraoperative and postoperative period until 48 hours postoperatively. Documentation includes number of patients receiving PRBC transfusions as well as amount of transfused packed red blood cells [ml]. Transfusion triggers are given in table 4 to standardize administration of PRBC.
Operation time [min]:	Time from skin incision to placement of last skin staple/suture.
Transection time [min]:	Time from beginning to end of liver transection.
Transection area [cm^2^]:	Surface area of the specimen.
Duration of postoperative hospital stay [days]:	Time from day of operation to day of discharge.
Duration of ICU stay [days]:	Time on the Intensive Care Unit (ICU). Patients' stay in the recovery room and Intermediate Care (IMC) unit exceeding 24 hours is considered as ICU stay.
Morbidaity:	Besides SAEs the following predefined complications are documented as AEs within IVC CLAMP: *Posthepatectomy haemorrhage*: Drop of haemoglobin >3 g/dl (after 6 hours after the end of surgery) or any *postoperative *transfusion of PRBCs for a falling haemoglobin and/or the need for reintervention (i.e. embolisation or re-laparotomy) *Postoperative biliary leakage*: Presence of bile fluid (bilirubin level > twice the serum level) in the abdominal cavity or drains > 48 hours beyond the end of surgery or the need for reintervention (i.e. interventional drainage or relaparotomy due to bile fluid collections or biliary peritonitis). *Further biliary complications*: Biliary complications such as postoperative biliary stricture detected via ERCP or MRCP *Intraabdominal fluid collection/abscess*: Intraabdominal fluid collection detected on any imaging modality (e.g. ultrasound, CT scan) associated with abdominal discomfort/pain or elevation of infectious parameters. *Posthepatectomy liver failure*: Postoperative deterioration of the liver's synthetic, excretory, and detoxifying functions ≥ day 5:
	• Increased INR or need of coagulation products (FFP, coagulation factors) to normalize the INR
	• Serum bilirubin ≥ twice upper limit of normal
	• Encephalopathy
	- *Pneumonia*: Pulmonary infection with evidence of increased infection parameters (CRP > 2 mg/dl and/or leukocytes > 10 000/ml) which are unlikely to be caused by a different pathologic process and evidence of pulmonary infiltrates on chest x-ray, requiring antibiotic therapy.
In-hospital mortality:	Death due to any reason within the patient's initial hospital stay.
Liver function:	ALT, AST, GGT, Quick's time/INR, bilirubin, and albumin preoperatively, intraoperatively and on postoperative days 1, 3 and 7.
Kidney function:	Serum creatinine and BUN preoperatively, intraoperatively and on postoperative days 1, 3 and 7.
Need for portal triad clamping:	Need for additional vascular control during actual parenchymal transection.
Haemodynamics and haemodynamic intolerance:	Heart rate, blood pressure and CVP are documented during liver transection. If fluid administration plus additional PRBCs prove insufficient in maintaining mean arterial pressure of at least 65 mmHg as does the use of up to 0.2 μg/kg body weight Noradrenaline every minute in infusion injection (i.e. 40 ml/h of Noradrenaline 1 mg/50 ml in a 70 kg body weight standard patient), executing anaesthetist may use up to two times 100 μg of Noradrenaline in bolus injections. If the executing anaesthetist uses Noradrenaline in bolus injections the patient is not considered haemodynamically stable any more. If the executing anaesthetist uses more than two times 100 μg of Noradrenaline in bolus injections the patient is considered in a life-threatening condition and treatment according to protocol is terminated. In this case the patient is analysed according to intention-to-treat.
Re-laparotomy:	Laparotomy within 30 days after the index operation.

The principal investigator may terminate the trial at any time in consultation with the key research associates and the biostatistician. Reasons for termination include incidence or severity of AEs indicating a potential health hazard caused by either study treatment and external evidence indicating likewise or requiring premature termination of the trial.

### Standardisation of treatments

#### Standardisation of anaesthesiological care

All patients are treated according to local standards of the Department of Anaesthesiology, University of Heidelberg:

#### Preoperative Care

Patients are asked to stop eating and drinking from midnight before surgery. Fluid loss due to fasting is not replaced before induction of anaesthesia.

Approximately 30 minutes before induction of anaesthesia sedation medication (Midazolam 3.5-7.5 mg) is administered adapted to body weight and individual demand at executing anaesthetist's discretion.

#### Immediate Preoperative Care

Once entered operating theatre patients are equipped with a 18 or 20 gauge peripheral venous catheter. Patients are treated with either combined neuraxial and general anaesthesia or general anaesthesia only. Decision for or against neuraxial anaesthesia is based on executing anaesthesiologist's clinical judgement and on patients' choice, i.e. written informed consent.

Neuraxial anaesthesia, i.e. thoracic epidural catheter is tested using Lidocain (60-80 mg). Analgesia is induced using Sufentanil (20 μg) and Ropivacain (40-50 mg). Over the last 30 minutes before testing and induction of analgesia up to 10 ml/kg body weight of any crystalloid fluid preparation are administered to replace relative blood loss (internal blood loss due to loss of vascular tone). Analgesia in maintained using Ropivacain (40-50 mg/h), again.

General anaesthesia is induced using either Thiopental (3-5 mg/kg body weight) or Propofol (1-2 mg/kg body weight) with additional Fentanyl (2-4 μg/kg body weight) or Sufentanil (0.2-0.4 mg/kg body weight) and Atracurium (0.5-0.6 mg/kg body weight), Cisatracurium (0.15 mg/kg body weight) or Rocuronium (0.6 mg/kg body weight). Anaesthesia is maintained using either Sevofluran (minimum alveolar concentration 0.8) or Desfluran (minimum alveolar concentration 0.8) with additional Fentanyl (up to 10 μg/kg body weight total) or Sufentanil (up to 1 μg/kg body weight total), again.

All drugs are administered adapted to body weight and individual demand within ranges given, in case of Ropivacain adapted to height and individual demand.

Immediately after successful induction of anaesthesia each patient is being equipped with:

• Gastric tube

• 20 gauge arterial catheter

• 14 gauge peripheral venous catheter

• Twin lumen central venous catheter

• In case of prior abdominal surgery transurethral catheter (in other cases a suprapubic catheter is placed intraoperatively)

#### Intraoperative care

Patients are randomised to either surgical or anaesthesiological intervention once entering the operation theatre using consecutively numbered, opaque and sealed envelopes. Surgical intervention consists of standard intravenous fluid therapy according to local standards based on current textbook opinion (Table 3) [17] and infrahepatic IVC clamping for the transection period. Anaesthesiological intervention consists of goal-directed intravenous fluid therapy according to local standards. Effect variable for goal-directed fluid therapy is CVP of no more than 5 mmHg. If goal of therapy cannot be attained using restricted intravenous fluid therapy only, further means necessary may be taken, namely administration of opioids within given ranges, administration of nitro compounds, or administration of diuretics at discretion of executing anaesthetist. Minimum requirement for intravenous fluid therapy is the administration of at least 1 ml/kg body weight + 40 ml of any crystalloid fluid preparation every hour, the algorithm for the administration of packed red blood cells (PRBC) is displayed in table 4.

**Table 3:** Fluid management according to current textbook opinion [17].

**Compensatory intravascular volume expansion (with epidural anaesthesia) [mL]**	**Compensatory intravascular volume expansion (without epidural anaestesia) [mL]**	**Deficit [mL/h]**	**Maintenance [mL/h]**	**Third space [mL/h]**	**Blood loss [mL]**
CrF: Body weight [kg] × 10 [mL/kg]	CrF: Body weight [kg] × 5-7 [mL/kg]	CrF: (Body weight + 40) [kg] × [mL/kg/h]	CrF: (Body weight + 40) [kg] × [mL/kg/h]	CrF: Body weight [kg] × 4-6 [mL/kg/h]	CrF: 3:1 [volume:volume] CoF: 1:1 [volume:volume]

mL = mililiters; h = hours; kg = kilogramms; CrF = Crystalloid solutions; CoF = Colloidal solutions

**Table 4:** Transfusion triggers during the IVC CLAMP Trial.

**Risk profile**	**Minimum hemoglobin (conversion factor 0.621)**
< 40 years **no **additional risk factors **no **organ function impairment	<5.5 g/dl or 3.4 mmol/l

≥ 40 years **no **additional risk factors **no **organ function impairment	<6 g/dl or 3.7 mmol/l

organ function impairment	<7 g/dl or 4.3 mmol/l

coronary artery disease with no ischemia carotid artery stenosis with no ischemia history of transient ischemic attack	<8 g/dl or 5.0 mmol/l

coronary artery disease with ischemia e.g. troponin elevation carotid artery stenosis with ischemia history of stroke	<10 g/dl or 6.2 mmol/l

If fluid administration plus additional RPBC prove insufficient in maintaining mean arterial pressure of at least 65 mmHg, the executing anaesthetist may use up to 2 ml of Akrinor^® ^(up to 200 mg Cafedrin/10 mg Theodrenalin) in bolus injections. In addition the executing anaesthetist may use up to 0.2 μg/kg body weight Norepinephrin every minute in infusion injection (i.e. 40 ml/h of Noradrenaline 1 mg/50 ml in a 70 kg body weight standard patient) and up to two times 100 μg Norepinephrin in bolus injections. If Noradrenaline is used in bolus injections the patient is not considered haemodynamically stable any more. The need for more than two times 100 μg Noradrenaline in bolus injections defines a life-threatening condition and treatment according to protocol is terminated (in this case the patient is analysed according to intention-to-treat).

#### Standardisation of surgical technique

Patients receive single shots of mezlocilline (4 g) and metronidazole (0.5 g) 30 minutes prior to incision. After being placed in supine position, patients are prepared and draped in a sterile fashion. Following a roof-top incision (with or without extension in the midline to the xiphoid), a reversed L-shaped incision from the xiphoid to the tip of the twelfth right rib, or a standard transverse abdominal incision, the abdomen is initially explored for extrahepatic disease. The falciform triangular ligament is mobilised and dissected to expose the hepatic veins and porta hepatis. To fully mobilise the liver, short hepatic and caudate veins from the IVC are clipped or ligated.

In general, transection of the liver parenchyma within IVC CLAMP is recommended to be performed as stapler hepatectomy to further standardise liver resection. However, should stapler hepatectomy not be feasible or considered to be potentially harmful to the patient, the ultrasonic dissection or the clamp-crushing technique may be chosen by the executing surgeon. The transection technique is documented and considered as a factor in covariance analysis and the interaction with intervention will be tested. While routine use of portal triad clamping is not recommended, the final choice to use portal is left at discretion of the executing surgeon and the need for portal triad clamping is documented as a secondary endpoint. Argon beam coagulation is applied to stop minor oozing once resection is completed and sealants may be used, if deemed necessary by the executing surgeon. After haemostasis is considered secure, easy-flow drains are placed in the subphrenic and subhepatic space and the abdomen is closed in a standardised manner.

### Trial interventions

#### Group A: Inferior vena cava clamping during hepatic resection

After laparotomy, the IVC is dissected free below the liver. Prior to the beginning of the actual transection period the IVC is briefly clamped with a vascular clamp to assess haemodynamic tolerance. In case the patient tolerates IVC clamping, transection of the parenchyma is started. Patients in this study group receive intravenous volume for maintenance of fluid homoeostasis according to local standard and current textbook opinion, i.e. intravenous fluid therapy sufficient to maintain normovolaemia judged by standard vital signs with special emphasis on CVP of 5-12 mmHg.

#### Group B: No clamping during hepatic resection

Patients in this study group undergo hepatic resection according to local standards of the Departments of General-, Visceral- and Transplant Surgery and the Department of Anaesthesiology, University of Heidelberg. Current practice requires CVP reduction by goal-directed intravenous fluid therapy, diuretics, and nitro compounds without any routine use of hepatic vascular control. Effect variable for goal-directed fluid therapy is CVP of no more than 5 mmHg. Furthermore, additional i.v. fentanyl, ropivacain via the epidural catheter and positive end-expiratory pressure (PEEP) to zero may be used to lower CVP below 5 mmHg.

### Study objectives and endpoints

It is the primary objective of this trial to assess and compare the amount of blood lost during hepatic resection in patients receiving surgical CVP reduction as compared to anaesthesiological CVP reduction without infrahepatic IVC clamping. In addition to blood loss a set of general as well as surgical variables are assessed.

Intraoperative blood loss [ml], i.e. the blood lost from skin incision until skin closure, was chosen as the primary endpoint. The amount of blood lost is measured from the amount of blood collected in the suction containers including blood squeezed from sponges and gauzes. The irrigation volume is subtracted. An independent study nurse documents intraoperative blood loss. Furthermore, an independent study nurse blinded to the allocated intervention documents all other endpoints.

Secondary endpoints are chosen in accordance with recently published randomised trials in the field of hepatic surgery [18-20]. The secondary endpoints are presented in Table 2.

### Trial implementation

Visits within IVC CLAMP are carried out by a study nurse blinded to the allocated treatment. Study visits and acquired data within IVC CLAMP are displayed in Table 5. The laboratory tests within IVC CLAMP are summarised in Table 6.

**Table 5:** Flow chart of the IVC CLAMP-Trial.

	**Screening**	**Intervention**	**Follow up**		
	
	**Visit 1** **(up to 30 days before surgery)**	**Visit 2** **(day of surgery)**	**Visit 3** **(POD 1)**	**Visit 4** **(POD 3)**	**Visit 5** **(POD 7 or day of discharge)**
Selection criteria Informed consent	X				

Medical history Demographics	X				

Physical examination	X				

Laboratory tests^1^	X		X	X	X

Intraoperative outcomes		X			

Postoperative outcomes					X

^1^For detailed description of laboratory test done see table 6. POD, Postoperative day

Past/current medical history: past/current medical/surgical history i.e. concomitant/underlying illness (indication for surgery)

Demographics: age [years], sex, ASA-class [26], NYHA-class [16]

Physical examination: height [m], body weight [kg], smoking habits [packs/day, pack years]

Intraoperative outcome measures: intraoperative blood loss, intraoperative PRBC transfusion, haemodynamics/haemodynamic instability, operation time, transection time, transection area, use of portal triad clamping, performed resection type/extent, transection technique, and mortality

Postoperative outcome measures: postoperative RPBC transfusion (until 48 h), postoperative complications/morbidity, duration of hospital stay, duration of ICU stay, mortality

**Table 6:** Laboratory tests performed within the IVC CLAMP Trial.

	**Parameters**	**Screening**	**Intervention**			**Follow up**	
		
		**Visit 1 (up to 30 days before surgery)**	**Visit 2 (after induction of anaesthesia prior to first incision)**	**Visit 2 (before resection)**	**Visit 2 (after resection, before end of surgery)**	**Visit 3 (POD 1)**	**Visit 4 + 5 (POD 3 + 7/day of discharge)**
Ventilation	Arterial oxygen saturation		X	X	X		
	Arterial power of hydrogen. Arterial partial pressure of oxygen and carbon dioxide. Base excess.		X	X	X		
Hematology	Small blood count (from whole blood).	X	X	X	X	X	X
Electrolyte metabolism	Sodium, potassium, calcium chloride.*	X	X	X	X	X	X
Kidney funtion	Creatinine, urea (from blood serum).	X	X	X	X	X	X
Liver damage/function	AST, ALT, gammaGT, Bilirubin (from blood serum).	X	X	X	X	X	X
Coagulation	Quick's time, INR, Fibrinogen, Antithrombin 3.	X	X	X	X	X	X
Infectious parameters	C-reactive protein (from blood serum).	X				X	X

*Measures of electrolyte metabolism (sodium, potassium, calcium, chloride) are determied from blood serum at visit 1, 3, 4 and 5. Intraoperatively, these measures are determined from arterial blood gas analysis.

### Sample size

The sample size is based on the primary efficacy endpoint variable and the primary analysis. Assuming a pooled standard deviation in blood loss of 458 ml (calculated from data taken from the literature [2,14] and internal observations at the Department of General, Visceral and Transplant Surgery, University of Heidelberg), 48 patients per group are needed to detect a difference of 280 ml with a significance α = 5% and a power of (1 - β) = 80% using two-sided t-test.

Taking into account the possible loss of power by using one additional factor in the analysis of covariance, another 8 patients per treatment group are randomised. The continuous covariates applied in this analysis do not decrease the power and therefore are not included in the sample size estimation.

As randomisation is carried out before laparotomy an intraoperative drop-out rate of 30% is assumed to consider possible intraoperative findings preventing hepatic resection (e.g. extrahepatic disease such as peritoneal carcinomatosis, technically inoperable disease, severe liver cirrhosis etc). Thus the total sample size accounts for 152 patients.

### Statistical analysis

Statistical methods are used to assess the quality of data, homogeneity of treatment groups, endpoints and safety of the two intervention groups. The confirmatory analysis is performed based on the intention-to-treat (ITT) patients and with respect to ITT principles.

Categorical data are summarised by means of absolute and relative frequencies (count and percent). Continuous data are presented by the following summary statistics: the number of observations, arithmetic mean, standard deviation, minimum, median and maximum. Wherever appropriate, results of these analyses are visualised (box-whisker plots or histograms).

The primary efficacy endpoint is intraoperative blood loss measured as previously described. The underlying two sided null-hypothesis is that both interventions lead to similar means in intraoperative blood loss:



The alternative hypothesis is that one intervention performs better than the other:



A confirmatory intention to treat analysis (2-sided test), including all patients as randomised, is calculated for the mean intraoperative blood loss between the two groups. Analysis of covariance techniques are used to detect possible treatment differences, with transection technique as additional factor and International Normalized Ratio (INR) as coagulation measure, the actual resection surface area and CVP during transection as continuous covariates. Secondary endpoints are analysed in an exploratory way, using appropriate statistical methods based on the underlying distribution of the data. Sensitivity analyses are calculated including different patient populations (per-protocol patients) and different statistical methods (t-test). All analyses are done using the SAS™ program Version 9.1.

### Randomisation and methods to minimise bias

Patients are randomly allocated to either study intervention to balance treatment groups for all known and unknown potentially confounding factors. The Institute for Medical Biometrics and Informatics (IMBI) generates a block randomisation list using SAS (SAS™ Version 9.1., SAS Institute Inc., Cary, USA). To reduce costs, patients are randomised using consecutively numbered opaque envelopes prepared and sealed by an independent study nurse. All patients screened as well as those included and randomised in the study are entered in a consecutive list. Envelopes are opened upon entrance of the patient in the operating room.

Executing surgeons are briefed on the trial interventions and permitted surgical techniques. IVC CLAMP is carried out in an interdisciplinary setting between surgeons and anaesthetists with the main goal to highly standardise trial interventions as well as concomitant care according to the study protocol (e.g. antibiotic prophylaxis, anaesthesia, etc.).

An independent study nurse is present in the operating room to monitor and document trial procedures and events. As blinding of participants in the operating room is not feasible, assessment of postoperative outcomes is carried out by a third party blinded to patients' treatment group as is the patient himself.

### Data management and quality assurance

A paper-based case report form (CRF) providing detailed instructions and definition of outcomes was designed for documentation within IVC CLAMP. An independent study nurse not involved in treatment and monitoring of the patients within the operating room enters all required data. The data are supposed to be entered as soon as possible, preferably at the day of a patient's visit and treatment, respectively. Treatments are documented in separate treatment logs. Standard adverse event forms that have been validated in previous trials are used for reporting of (serious) adverse events.

### Ethical and legal considerations

IVC CLAMP is carried out according to the current Declaration of Helsinki (sixth revision, 2008), the principles of "Good Clinical Practice" (GCP), and the Federal Data Protection Act.

The clinical trial protocol, patient information and informed consent sheet have been approved by the independent ethics committee of the University of Heidelberg, Medical School. IVC CLAMP is registered at the ClinicalTrials.gov protocol registration system . The assigned identification number is NCT00732979.

All patients are instructed on the trial's nature, scope, and possible consequences by a physician on the day prior to surgery and patients' written informed consent has to be obtained for patient enrolment. Participation in IVC CLAMP is voluntary and patients can withdraw their consent at any time without any negative consequence for their further treatment.

Patients' personal data are subject to the legal requirement ensuring confidential medical communication and the regulations of the Federal Data Protection Act ("Bundesdatenschutzgesetz"). Patient data are assessed pseudo-anonymously and transferred and analysed anonymously. No person not involved in the trial has access to patient data.

## Discussion

IVC CLAMP represents a RCT to evaluate efficacy and safety of infrahepatic IVC clamping in comparison to anaesthesiological methods for CVP reduction in hepatic resections. CVP reduction carried out by the anaesthesiologist was introduced in the late 1990's as an effective technique to reduce blood loss during transection of the liver parenchyma [6,7]. By restricted intravenous fluid administration and if required additional application of nitro-compounds and diuretics CVP can be reliably lowered to values below 5 mmHg which results in less bleeding from the hepatic veins. Based on these results CVP reduction has been widely adopted as part of the perioperative management of patients undergoing liver resection as indicated by various randomised studies in the field of liver surgery [13,18,21]. Dehydration might, however, increase patients' risk for microperfusion deficits with consecutive organ dysfunction as well as for haemodynamic instability and there is still very limited data on safety of patients undergoing liver resection under the condition of anaesthesiological CVP reduction. Against the background of relevant morbidity associated with hepatic resections [1,3,22,23], a critical evaluation of currently performed CVP reduction and alternative techniques to lower CVP are required.

The present trial compares CVP reduction via infrahepatic IVC clamping to common anaesthesiological CVP reduction, which is primarily carried out by restricted intravenous fluid administration and application of diuretics or nitro-compounds without any type of vascular clamping. This study design allows assessing efficacy and safety of infrahepatic IVC clamping as well as a critical evaluation of anaesthesiological CVP reduction as the current standard of practice.

In accordance with various previous RCTs in the field of liver surgery intraoperative blood loss was chosen as primary endpoint of the present trial. The clinical relevance of this variable has been demonstrated by several studies that showed intraoperative blood loss to be an independent predictor of perioperative outcome and reduction of intraoperative bleeding is therefore a primary goal for surgeons as well as anaesthetists [3,5]. We deliberately chose entire intraoperative blood loss as opposed to blood loss during transection of the parenchyma as primary outcome measure, as we intended to compare the two strategies of CVP reduction to each other. As fluid restriction starts right away and thus affects the entire operation prior to actual liver transection, it may have an impact on patients' blood loss through the entire duration of the operation.

This trial is designed as a randomised controlled observer and patient-blinded two-group parallel trial in an attempt to minimise bias and by this to enable most valid results. Due to the fact that in the study group undergoing anaesthesiological CVP reduction restriction of intravenous fluid administration has to be started in advance, randomisation in the present trial has to be carried out prior to abdominal incision. Most patients undergo hepatectomy for malignant disease. Commonly, no resection is carried out in case exploration of the liver and abdominal cavity reveals extrahepatic disease, peritoneal dissemination, and non-resectable hepatic lesions, respectively. To consider this proportion of patients that actually does not receive hepatectomy, an additional 30% was added to the calculated sample size.

The present trial was designed with the intention to minimise bias and by this to generate high-quality data that help clinicians in their decision-making for the optimal technique to reduce CVP during hepatectomy. Outcomes are assessed by an independent study nurse who is not involved in the design and analysis of the trial. Furthermore, all outcomes have been defined a priori (Table 2). Besides intraoperative blood loss transfusion of packed red blood cells is known to adversely affect patients' outcome [5]. To enable valid comparison of the need for blood transfusion between patients in both study arms, standardised transfusion triggers were established.

Within IVC CLAMP transection of the liver parenchyma by stapler hepatectomy is recommended. However, the final choice is at discretion of the executing surgeon considering patients' safety and feasibility of resection. To limit the number of applied methods, the three most common techniques (i.e. clamp-crush technique, stapler hepatectomy, and ultrasonic dissection) are allowed within the present trial. We are confident that allowance of three transection techniques does not confound the results, as currently available evidence does not favour one technique regarding intraoperative blood loss [24,25]. Furthermore, this approach increases external validity of the trial. However, to control for potential bias the applied transection technique is documented and considered as a factor in covariance analysis.

In conclusion, the present randomised controlled observer and patient-blinded two-group parallel trial compares the technique of infrahepatic IVC clamping to common anaesthesiological methods for reduction of CVP during hepatectomy. The results help to assess efficacy and safety of infrahepatic IVC clamping as a routine technique to lower CVP. Furthermore, they provide a critical appraisal of the safety of anaesthesiological CVP reduction and by this a potential strategy to reduce current morbidity associated with hepatic resection.

## Competing interests

The authors declare that they have no competing interests.

## Authors' contributions

This study was designed by NNR, JBZ and JW who also wrote the article. TB performed the sample size calculation and planned the statistical analyses. MK, TS, HE, CR, MAW and MWB are involved in trial implementation and critically revised the manuscript. All authors have read and approved the manuscript.
